# Dipsomania

**Published:** 1875-04

**Authors:** 


### DIPSOMANIA.

Some extraordinary instances of the insatiate desire, or rather morbid impulse, to drink are mentioned by Dr. George Burr, in a recent paper on the "Insanity of Inebriety." Dr. Bush records a case of an habitual drunkard in Philadelphia, who, when strongly urged by one of his friends to leave off drinking, replied, "Were a keg of rum in one corner of a room, and were a cannon constantly discharging balls between me and it, I could not refrain from passing before that cannon in order to get at the rum." One of the cases described by McNish, in his "Anatomy of Drunkenness," also illustrates this feature. A friend of the subject of it painted to him the distresses of his family, the loss of his business and character, and the ruin of his health, to which he replied, "My good friend, your remarks are just; they are indeed too true; but I can no longer resist temptation. If a bottle of brandy stood at one hand, and the pit of hell yawned at the other, and I were convinced that I would be pushed in as sure as I took one glass, I could not refrain." The late Professor R. D. Mussey, of Cincinnati, relates another case: "A few years ago a tippler was put into an almshouse in this State. Within a few days he had devised various expedients to procure rum, but failed. At length, however, he hit upon one which was successful. He went into the wood-yard of the establishment, placed one hand upon the block, and, with an axe in the other, struck it off at a single blow. With the stump raised and streaming, he ran into the house and cried, 'Get some rum! get some rum! my hand is off.' In the confusion and bustle of the occasion a bowl of rum was brought, into which he plunged the bleeding member of his body, then, raising the bowl to his mouth drank freely, and exultingly exclaimed, 'Now I am satisfied!'" Dr. J. E. Turner relates a case of a gentleman who, while under treatment for inebriety, during four weeks secretly drank the alcohol from six jars containing morbid specimens. On asking him why he had committed this loathsome act he replied, "Sir, it is as impossible for me to control this diseased appetite as it is for me to control the pulsations of my heart."

